# Horner’s Syndrome Following Anterior Cervical Spinal Surgery: A Case Report

**DOI:** 10.22336/rjo.2026.60

**Published:** 2026

**Authors:** Merve Cetin, Tuncay Kusbeci, Omer Karti

**Affiliations:** 1 Izmir City Hospital, Izmir, Turkey; 2 University of Health Sciences, Izmir Faculty of Medicine, Izmir, Turkey; 3 Dokuz Eylül University, Izmir, Turkey

**Keywords:** Horner’s syndrome, myosis, ptosis, HS = Horner’s Syndrome, RAPD = Relative Afferent Pupillary Defect, ACDF = anterior cervical discectomy and fusion

## Abstract

**Objectives:**

Horner syndrome is a well-known but rare complication of anterior-approach cervical spinal surgery. Cervical sympathetic chain injury results in ipsilateral pupillary ptosis, miosis, and facial anhidrosis. In this case report, we aimed to present a case of right-sided Horner syndrome (blepharoptosis, miosis, and anhidrosis) that developed postoperatively after anterior cervical discectomy for cervical disc herniation.

**Case report:**

A 44-year-old woman presented to the ophthalmology clinic with complaints of right-sided eyelid ptosis. Her medical history revealed that she had undergone anterior cervical discectomy at the C3-C4 disc level for cervical disc herniation 3 weeks prior. Best-corrected visual acuities were 20/25 on the right and 20/20 on the left. Intraocular pressure was measured as 15 on the right and 14 mm Hg on the left.

**Results:**

Marked ptosis and miosis, obscuring the pupillary area on the right, were observed. Marked anisocoria was present. Pupillary diameters measured 2 mm on the right and 5.5 mm on the left under scotopic conditions, and 1.5 mm on the right and 3 mm on the left under photopic conditions. No RAPD was detected. At the patient’s follow-up examination one week later, 0.5% aproclonidine was administered to both eyes. Following the test, the right pupil was more mydriatic than the left, and a significant decrease in ptosis was observed.

**Discussion:**

Horner syndrome is a rare complication of anterior cervical spinal surgery caused by sympathetic chain injury. Persistent symptoms may indicate severe or permanent damage to the sympathetic pathway. Careful dissection and limited retraction may help minimize this risk.

**Conclusions:**

Horner Syndrome is a very rare complication that occurs due to ipsilateral sympathetic nerve damage after an anterior cervical approach. Intraoperative assessment of this important anatomic structure is crucial.

## Introduction

Horner’s syndrome (HS) is a well-known but rare syndrome characterized by ipsilateral ptosis, miosis, and facial anhidrosis. It can occur due to injury to the cervical sympathetic nerve. It can develop as a complication during surgery or as a result of mechanical trauma from retraction, particularly in anterior-approach cervical spinal surgeries, as the sympathetic nerve runs in proximity to the longus colli muscle, particularly at the C6 level [1,2]. Frequent retraction, dissection, or cauterization of the muscle during surgery makes the cervical sympathetic trunk, located immediately adjacent to this muscle, vulnerable to trauma [3].

A multicenter retrospective study screened 8,887 patients undergoing anterior cervical spine surgery and identified postoperative HS in five patients (0.06%). The sympathetic trunk was observed to be more susceptible to trauma, particularly during surgery at the C5-C6 levels, and most patients achieved partial recovery without additional treatment [4]. HS is usually transient, with symptoms resolving within a few weeks; however, if there is severe trauma to the sympathetic chain, recovery may be delayed or permanent [5].

In this case report, we aimed to present a case of right-sided HS (blepharoptosis, miosis, anhidrosis) that developed postoperatively after an anterior cervical discectomy performed for cervical disc herniation.

## Case report

A 44-year-old woman presented to the ophthalmology clinic complaining of right eyelid ptosis. Her medical history revealed that she had undergone anterior cervical discectomy (anterior cervical discectomy) at the C3-C4 disc level three weeks prior for a herniated cervical disc. Her symptoms began after surgery, and there was no change in ptosis over time. A preoperative cervical MRI revealed a central extruded herniation in the posterior contour at the C4-C5 level (**Fig. 1**). The patient had undergone surgery three years prior for a herniation at the C5-C6 level and underwent further surgery due to the recurrence of numbness in her arm.

**Fig. 1:**
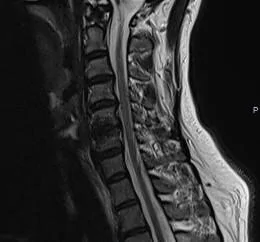
Central extruded herniation at C4-C5 on preoperative cervical MRI

On ophthalmologic examination, best-corrected visual acuity was 20/25 in the right eye and 20/20 in the left eye. Intraocular pressure was measured as 15 mm Hg in the right eye and 14 mm Hg in the left eye. There was 2 mm of ptosis and miosis covering the pupillary area in the right eye. Marked anisocoria was present. Pupillary diameters were measured as 2 mm on the right and 5.5 mm on the left under scotopic conditions and 1.5 mm on the right and 3 mm on the left under photopic conditions. No RAPD was detected. Anterior segment examination revealed no additional pathology. Fundus examination was normal bilaterally (**Fig. 2**).

**Fig. 2:**
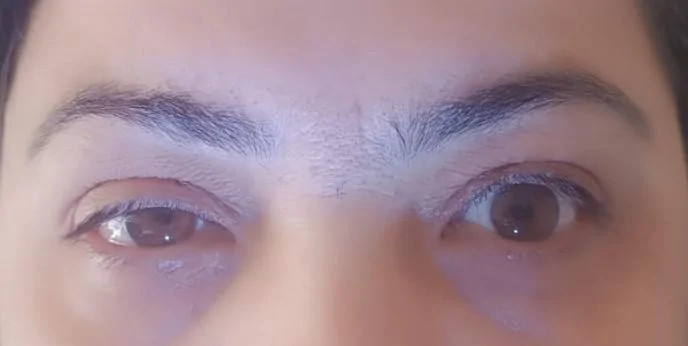
Right-sided ptosis with miosis compared with the left eye

At the patient’s follow-up visit one week later, 0.5% apraclonidine was administered to both eyes. Following the test, the right pupil was more mydriatic than the left, and a significant decrease in ptosis was observed (**Fig. 3**). No changes were observed in the patient’s findings at the 3rd and 6th month follow-up visits.

**Fig. 3:**
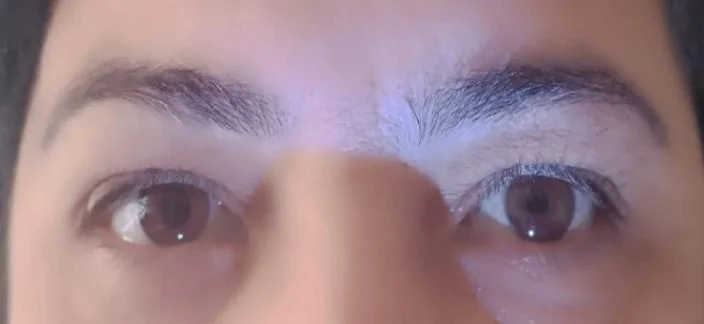
Improvement of right-sided ptosis and relative mydriasis in the right pupil after 0.5% apraclonidine

## Discussion

HS is divided into three groups based on the level of sympathetic nerve pathway involvement: central, preganglionic, and postganglionic. The central form is caused by first-degree neuronal damage, while the preganglionic form is the most common and is typically associated with apical lung pathologies. The postganglionic form occurs when fibers following the superior cervical ganglion are affected, and it is most often due to vascular, traumatic, or infectious causes [6,7]. Particularly during anterior cervical discectomy and fusion (ACDF), the risk of injury at the C5-C6 level increases because the sympathetic chain courses more medially and laterally than the longus colli muscle, and HS may develop. In the literature, the overall morbidity rate associated with ACDF ranges from 13.2% to 19.2%, and the incidence of HS has been reported as between 0.06% and 1.1% [8].

Pupil dilation is slowed in HS. This delay can be detected by pupillography and aids in diagnosis. The topical cocaine test, historically widely used for diagnosis, has now been replaced by 0.5% apraclonidine. Apraclonidine has similar sensitivity and specificity to the cocaine test and is primarily an alpha-2 adrenergic agonist. In HS, denervation hypersensitivity develops at alpha-1 receptors of the pupillary dilator muscle, reversing anisocoria [9,10]. Morales et al. reported that 1% apraclonidine can be used to diagnose HS. At the same time, Brown et al. demonstrated that the less expensive and more readily available 0.5% apraclonidine can be used similarly [11,12]. In our patient, after the administration of 0.5% apraclonidine, the affected right pupil became more mydriatic, and the ptosis decreased, confirming the diagnosis.

In our case, anterior cervical spinal surgery was performed at the C3-C4 level, and the patient had a history of surgery at the C5-C6 levels three years prior. Although no sympathetic chain injury was observed during surgery, it is possible that the chain was damaged during retraction. No improvement was observed at the six-month follow-up. This suggested that the sympathetic chain might have suffered more severe and permanent damage. Traynelis et al. reported that the cervical regions most frequently associated with HS after cervical spinal surgery were C5 and C6, followed by C7, C4, and C3 [4]. They observed complete recovery in 40% of patients, partial recovery in 20%, and no recovery in 20%. Umimura et al. presented two cases after lower cervical spinal surgery: in the first case, a 58-year-old woman developed right-sided miosis and anhidrosis, which persisted for more than 15 years; in the second case, a 40-year-old woman developed ptosis and miosis that lasted approximately four months [13]. Jiawen et al. reported that a 48-year-old woman developed HS approximately 48 hours after C5–C6 and C6–C7 ACDF; symptoms began to improve within two weeks and resolved completely by 12 weeks [14]. They emphasized that factors such as duration of surgery, type of anesthesia, and level of herniation may increase the risk of this complication.

## Conclusion

HS is a rare complication that can occur after the anterior cervical approach due to ipsilateral sympathetic chain injury. It is crucial to consider the presence of this important anatomic structure during surgery. Furthermore, it is important to inform patients about this complication as a potential risk before surgery.
